# Cs-Doped WO_3_ with Enhanced Conduction Band for Efficient Photocatalytic Oxygen Evolution Reaction Driven by Long-Wavelength Visible Light

**DOI:** 10.3390/molecules29133126

**Published:** 2024-06-30

**Authors:** Dong Li, Siyu Tian, Qiuhua Qian, Caiyun Gao, Hongfang Shen, Fei Han

**Affiliations:** 1 School of Materials Science & Engineering, North Minzu University, Yinchuan 750021, China; tiansiyu0907@163.com (S.T.); 13020598307@163.com (Q.Q.); shen_hongfang@nun.edu.cn (H.S.); hanfei@nun.edu.cn (F.H.); 2 National and Local Joint Engineering Research Center of Advanced Carbon-Based Ceramics Preparation Technology, Yinchuan 750021, China; 3 Chemical Science and Engineering College, North Minzu University, Yinchuan 750021, China; caiyun-gao@nun.edu.cn

**Keywords:** Cs-doped, tungsten trioxide, oxygen vacancy, band energy, oxygen evolution reaction

## Abstract

Cesium doped WO_3_ (Cs-WO_3_) photocatalyst with high and stable oxidation activity was successfully synthesized by a one-step hydrothermal method using Cs_2_CO_3_ as the doped metal ion source and tungstic acid (H_2_WO_4_) as the tungsten source. A series of analytical characterization tools and oxygen precipitation activity tests were used to compare the effects of different additions of Cs_2_CO_3_ on the crystal structure and microscopic morphologies. The UV–visible diffuse reflectance spectra (DRS) of Cs-doped material exhibited a significant red shift in the absorption edge with new shoulders appearing at 440–520 nm. The formation of an oxygen vacancy was confirmed in Cs-WO_3_ by the EPR signal, which can effectively regulate the electronic structure of the catalyst surface and contribute to improving the activity of the oxygen evolution reaction (OER). The photocatalytic OER results showed that the Cs-WO_3_-0.1 exhibited the optimal oxygen precipitation activity, reaching 58.28 µmol at 6 h, which was greater than six times higher than that of WO_3_-0 (9.76 μmol). It can be attributed to the synergistic effect of the increase in the conduction band position of Cs-WO_3_-0.1 (0.11 V) and oxygen vacancies compared to WO_3_-0, which accelerate the electron conduction rate and slow down the rapid compounding of photogenerated electrons–holes, improving the water-catalytic oxygen precipitation activity of WO_3_.

## 1. Introduction

Hydrogen energy converted from abundant solar energy is believed to be a sustainable green fuel to replace traditional fossil fuel due to its being pollution-free and low-cost [1]. Hydrogen production and oxygen production can be generated simultaneously by photocatalytic water splitting, which results from a hydrogen evolution reaction (HER) and OER [2]. Compared with the HER, the OER, as a four-electron transfer reaction, requires higher photon energy to overcome the reaction kinetic barriers and it is considered to be the rate-determining step in the entire water splitting process [3,4]. Currently, IrO_2_ and RuO_2_ have been widely used as photocatalysts for the OER due to their high catalytic activities [5,6]. However, these noble metals are scarce and costly, limiting their large-scale application for water splitting. Therefore, the development of an advanced photocatalyst based on low-cost and earth-abundant materials with highly efficient acceleration of the reaction dynamic and lowering of the energy barrier of OER is core issue for photocatalytic water splitting.

In recent years, some nanostructured semiconductor photocatalysts with lower OER overpotentials, including WO_3_, Fe_2_O_3_, BiVO_4_, TaON, BaZrO_3_-BaTaO_2_N and so on, have been explored [7,8,9,10,11]. Among these compounds, WO_3_ has attracted immense attention owning to its suitable bandgap (2.4–2.8 eV) [12,13], earth abundance, good chemical stability in strongly acidic media and its thermodynamically suitable valence band positions for OER. Nevertheless, WO_3_ suffers from limited visible-light response capability (*λ* < 460 nm), resulting in poor OER kinetics. Therefore, it is urgent to develop a WO_3_ photocatalyst with efficient light absorption at longer wavelengths in the visible region. Nonmetal element doping as one of the most effective strategies to improve the visible-light absorption of WO_3_ has been confirmed due to the formation of an intermediate level between the conduction band (CB) and valence band (VB) of WO_3_, resulting in the decrease in the band gap [14,15]. Also, metal element doping has already been used as a strategy to promote the visible-light absorption of WO_3_ because the conduction band position of WO_3_ can be adjusted to narrow the bandgap [16]. 

At present, investigations on doped WO_3_ by metal doping (Ti, Fe, Co, Ni, Cu, Zn, Yb, etc.) [17,18,19,20,21,22], rare earth metal doping [23] and nonmetal element doping (N, C, S, P) [24,25,26,27] have reported mostly to improve not only light absorption at longer wavelengths but also the photocatalytic performance for OER. Noted that cesium-doped WO_3_ has rarely been reported yet, although photocatalysts containing alkali metal cesium ions (Cs^+^) such as Cs_0.3_WO_3_, CsBi_2_Nb_5_O_16_, Cs_3_PW_12_O_40_, Cs_2_V_4_O_11_, Cs-doped *α*-Bi_2_O_3_ and Cs-doped TiO_2_ have been reported [28,29,30,31,32,33]. The reported results indicated that the photocatalytic activities could be enhanced by the introduction of Cs^+^ due to the low ionization energy of Cs in the lattices. 

Herein, we report the first synthesis of Cs-doped WO_3_ (Cs-WO_3_) photocatalysts with Cs^+^ doping into the WO_3_ lattice through the hydrothermal method using cesium carbonate (Cs_2_CO_3_) as the Cs ion source. The Cs-WO_3_ was responsive to visible light of *λ* ≤ 520 nm, which give a red shift of 80 nm compared with that of pristine WO_3_ (440 nm). The photocatalytic activities of Cs-WO_3_ for OER were significantly enhanced compared with pristine WO_3_, because the CB potential of WO_3_ is significantly increased through cesium doping, while introducing more lattice defects and oxygen vacancies, thereby improving the conductivity of the semiconductor. In particular, there is an extremely significant mutually dependent relation between the addition of the Cs source and the photocatalytic activities of Cs-WO_3_ for OER.

## 2. Results and Discussion

### 2.1. Characterization and Influencing Factors of Structure of Cs-WO_3_

**Figure 1:**
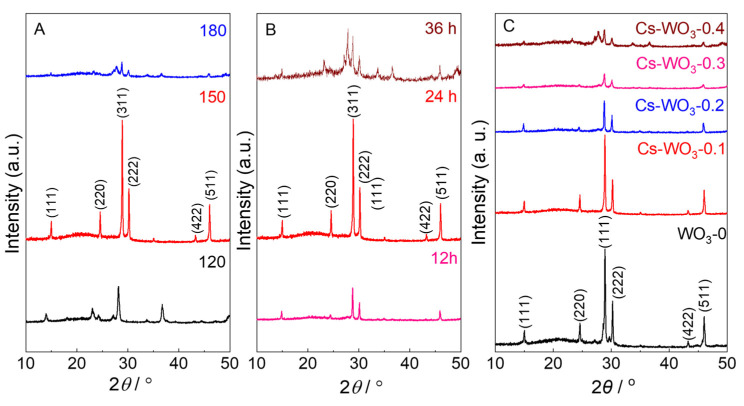
The XRD patterns of Cs-WO_3_-0.1 samples prepared at (**A**) different temperatures and (**B**) different reaction times. (**C**) The XRD patterns of the pure WO_3_ and Cs-WO_3_ samples prepared with different doping concentrations of Cs^+^ ions.

The results of the influencing factors on the structure of Cs-WO_3_-0.1 are shown in Figure 1. Figure 1A exhibits the XRD patterns of the samples prepared at different solvothermal temperatures from 120 to 180 °C. The XRD pattern prepared at 120 °C agreed with that of orthorhombic H_2_WO_4_ (JCPDS no. 01-084-0886). When the solvothermal temperature was 150 °C, it can be clearly observed that the peaks at 2*θ* = 15.0°, 24.6°, 28.9°, 30.3°, 43.2° and 46.1° correspond to the (111), (210), (311), (222), (422) and (511) plane, respectively, which can be assigned to a cubic WO_3_·0.5H_2_O crystalline phase (PDF # 01-084-1851). The peaks intensities of the Cs-WO_3_-0.1 decreased when increasing the temperature to 180 °C, and meanwhile the peaks at 27.2° and 27.8° assigned to the Cs_2_W_6_O_19_ compound (PDF # 01-045-0522) were formed. The XRD patterns of Cs-WO_3_-0.1 prepared at 150 °C for different reaction times of 12~36 h are exhibited in Figure 1B. The peak intensities of Cs-WO_3_-0.1 increased with the reaction time from 12 to 24 h, and thereby decreased above 24 h due to the formation of the Cs_2_W_6_O_19_ compound. The XRD results suggest that the optimum preparation conditions of Cs-WO_3_-0.1 are a solvothermal temperature of 150 °C and a reaction time of 24 h, which were employed in our experiments. As shown in Figure 1C, the XRD patterns of the WO_3_-0 and Cs-WO_3_ samples show the characteristic peaks of the cubic WO_3_·0.5H_2_O crystalline phase (PDF # 01-084-1851). However, the peaks of the Cs_2_W_6_O_19_ compound began to be seen in the Cs-WO_3_-0.5 alongside the main peaks of the cubic phase WO_3_·0.5H_2_O while overdoping with Cs^+^ ions. In Figure 1B,C, obvious shifts of XRD peaks are observed, which are ascribed to the existence of structural strain [34]. Additionally, compared with the WO_3_-0, the crystallinity of the Cs-WO_3_ samples decreased with increasing the doping concentration of Cs ions. This may be due to the disruption of the crystal structure of WO_3_, when the Cs ions with larger ionic radii (1.67 Å) are doped into the WO_3_ lattice, resulting in the formation of a distorted structure.

The average crystal grain size was calculated from the (311) peak (2*θ* = 28.9˚) of cubic WO_3_·0.5H_2_O according to Scherrer’s equation [35], as shown in Table 1. The crystallite diameter of the WO_3_-0 (23.6 nm) was larger than those of Cs-WO_3_-0.1 (22.6 nm), Cs-WO_3_-0.3 (20.9 nm) and Cs-WO_3_-0.5 (19.1 nm). The calculated lattice parameters and average crystalline size of WO_3_ were decreased (Table 1) by Cs doping. The results indicate that Cs-doped WO_3_ has a strong restraining function with the increase in crystal size owning to the dopant cations Cs^+^ preventing the growth of crystal grains in the nanoparticles [36].

**Figure 2:**
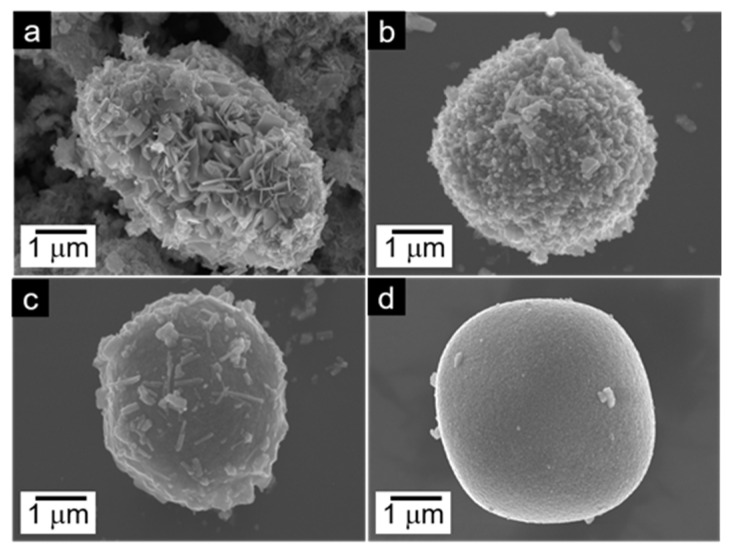
The SEM images of (**a**) WO_3_-0, (**b**) Cs-WO_3_-0.1, (**c**) Cs-WO_3_-0.3, (**d**) Cs-WO_3_-0.5.

As shown in Figure 2a, the elliptical-like WO_3_-0 is composed of nanosheets and nanoparticles with a major axis of about 55 μm and a semimajor axis of 40 μm. Notably, the Cs-WO_3_-0.1 is made up of a microsphere of about 41 μm in diameter which mainly consists of nanoparticles and nanorods (Figure 2b). The diameter of the microsphere (43 μm) increased and the amount of nanorods gradually decreased with the increasing doping amount of Cs^+^ (Figure 2c). A smooth microsphere with a diameter of 45 μm was formed for the Cs-WO_3_-0.5 (Figure 2d), suggesting that the formation of micrometer spheres mainly relies on self-assembly effects. As shown in Table 1, it was found that the specific surface area of the sample strongly depends on its morphology. The Cs-WO_3_-0.1 possessed a higher specific surface area (16.1 m^2^/g) relative to those of WO_3_-0 (9.2 m^2^/g) and Cs-WO_3_-0.3 (10.6 m^2^/g) owing to the formation of nanorods on the surface of the microsphere, which is beneficial to form more accessible active sites for the OER.

**Figure 3:**
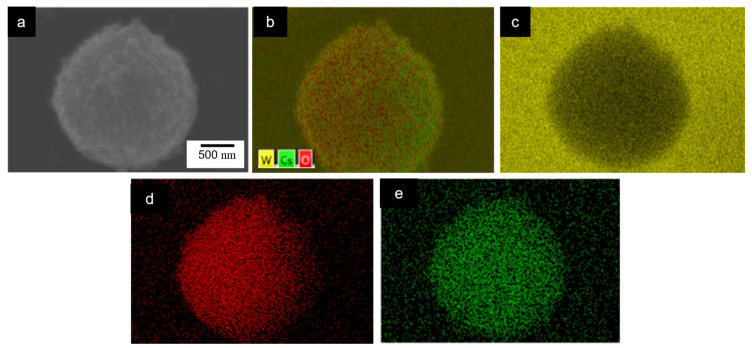
The SEM_EDX element distribution mapping images of the (**a**) Cs-WO_3_-0.1; (**b**) layered W, O and Cs; (**c**) W; (**d**) O; (**e**) Cs.

The elemental maps of the EDX for the Cs-WO_3_-0.1 are shown in Figure 3. The mapping signals of the W and O (Figure 3c,d) and of the Cs (Figure 3e) were detected. The results showed a uniform distribution of Cs, O and W, in good chemical agreement with Cs-WO_3_.

**Figure 4:**
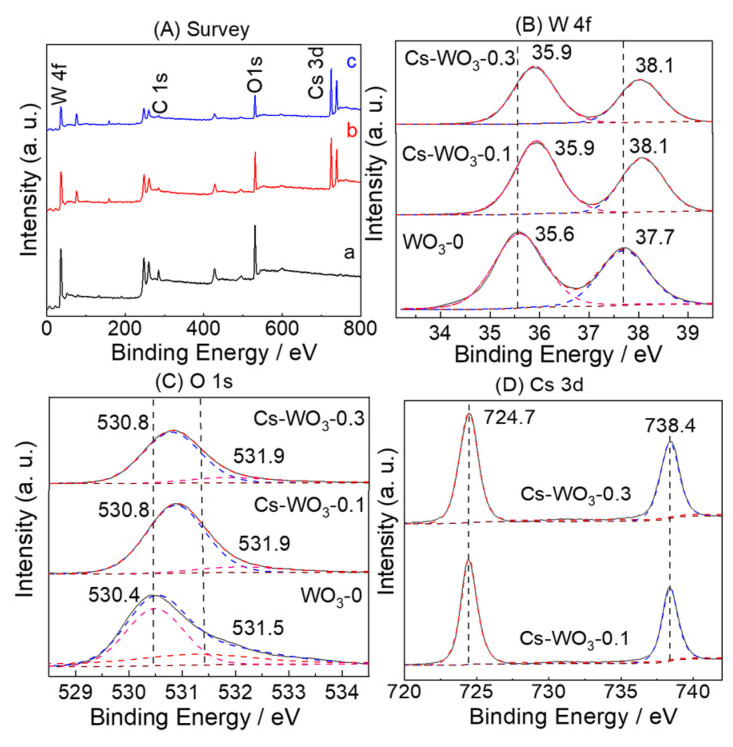
(**A**) The XPS survey spectra of (a) WO_3_-0, (b) Cs-WO_3_-0.1 and (c) Cs-WO_3_-0.3; (**B**) W 4f; (**C**) O 2p; and (**D**) Cs 3d regions.

The chemical composition and valence states of Cs-WO_3_ samples were investigated by XPS. The spectra were calibrated with the C 1s peak as reference. As shown in Figure 4A, the XPS survey spectrum of WO_3_-0 (a) depicts that no other impurity phases were detected except W and O elements. W, O and Cs elements were co-present in the Cs-WO_3_-0.1 (b) and Cs-WO_3_-0.3 (c). In Figure 4B, the high-resolution XPS spectrum of W 4f exhibits two peaks at 37.7 eV and 35.5 eV that are, respectively, ascribed to the spin–orbit doublet of W 4f_5/2_ and W 4f_7/2_, respectively, for a W^6+^ state in WO_3_ [37]. The XPS spectra of W 4f for Cs-WO_3_-0.1 and Cs-WO_3_-0.3 (Figure 3b) exhibit two characteristic peaks at 38.1 eV and 35.9 eV, corresponding to 4f_5/2_ and W 4f_7/2_ of the WO_3_ lattice, respectively. As shown in Figure 3c, the binding energy at 531.5 eV and 530.4 eV in the XPS spectrum of O 1s for the WO_3_-0 can be assigned to the H_2_O and lattice oxygen, respectively [38]. The main peaks in the high-resolution O 2p spectra for the Cs-WO_3_-0.1 and Cs-WO_3_-0.3 located at 530.8 eV can be assigned to the lattice oxygen of the W-O bond in the crystalline WO_3_. The banding energies at 531.9 eV correspond to the adsorbed oxygen ions and hydroxyl groups on the surface, respectively. The ratios of adsorbed O increased to 12.7% and 13.5% in Cs-WO_3_-0.1 and Cs-WO_3_-0.3 due to the surface oxygen vacancies, while it was 10.3% in WO_3_. This can serve as indirect evidence for the presence of oxygen vacancies. The high-resolution XPS spectra of the Cs element for both Cs-WO_3_-0.1 and Cs-WO_3_-0.3 (Figure 3d) exhibited two peaks at 738.4 eV and 724.7 eV, which are assigned to the spin orbits of Cs 3d_3/2_ and Cs 3d_5/2_, respectively [32,39]. Notably, the positive shifts of 0.4 eV and 0.8 eV in W 4f and O 1s for Cs-WO_3_ can be seen after Cs^+^ doping. This is due to the occurrence of ion exchange on the WO_3_ surface, which also confirmed that the Cs was successfully introduced into the lattice of WO_3_ and formed W-Cs bonds in the doped samples The positive shifts improve the PEC performance of WO_3_. Furthermore, it can be seen that the peak intensities of W 4f and O 1s for Cs-WO_3_ decreased with increasing the doped contents of Cs element because of the replacement of W^6+^ by Cs^+^ leading to the contamination of oxygen. Generally, the larger the ionic radius is, the more difficult it is for doping to occur due to the requirement of high formation energy. Therefore, the replacement of W^6+^ by Cs^+^ is more favorable than replacing O^2^- with Cs^+^. 

**Figure 5:**
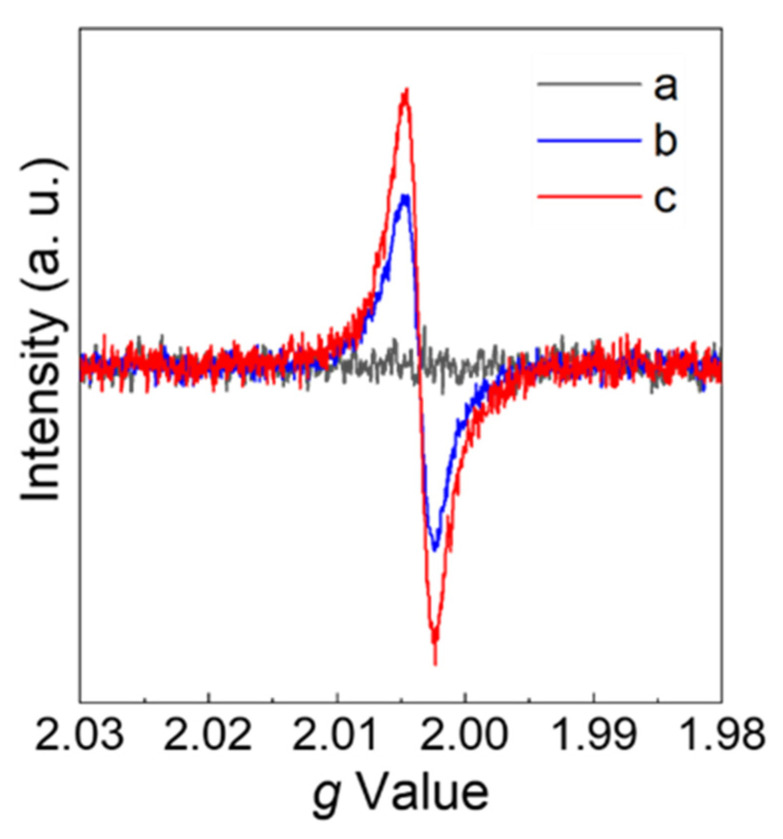
The EPR spectra of (**a**) WO_3_-0, (**b**) Cs-WO_3_-0.1 and (**c**) Cs-WO_3_-0.3 at 103 K in liquid N_2_.

In situ electron paramagnetic resonance (EPR) measurements were carried out to further prove the existence of surface oxygen vacancies and investigate their properties. No signal was detected for the WO_3_-0; however, Cs-WO_3_ samples exhibited relatively stronger EPR peaks intensity at g ≈ 2.002 under the same conditions, as shown in Figure 5. The higher the doped amounts of Cs, the stronger the signal intensity. These results agreed with results reported previously, confirming the presence of surface oxygen vacancies on Cs-WO_3_ samples [40,41]. The oxygen vacancies may be beneficial to improve the photocatalytic performance of Cs-WO_3_.

### 2.2. The Optical Properties of Cs-WO_3_

**Figure 6:**
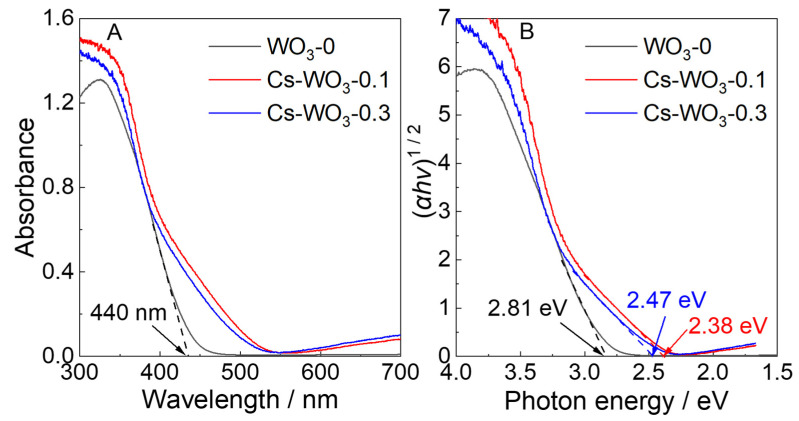
(**A**) UV–visible DRS and (**B**) Tacu plots based on UV–visible DRS of (black) WO_3_-0, (red) Cs-WO_3_-0.1 and (blue) Cs-WO_3_-0.3.

Figure 6 shows the UV–visible DRS spectra in the range from 300 to 700 nm and Tauc plots of WO_3_-0 and Cs-WO_3_ samples, respectively. As shown in Figure 6A, WO_3_-0 can only absorb light below 440 nm. However, a significant red shift in the absorption edge with new shoulders appearing at 440–520 nm can be seen for Cs-WO_3_ samples. Absorption above 600 nm was observed for Cs-WO_3_ samples because of the formation of an oxygen defect caused by doping [42], in contrast to negligible absorption for neat WO_3_, which is consist with the results of XPS and EPR. Furthermore, Figure 6B shows the Tauc plots based on DRS data, in which two different slopes are observed for the Tauc plots of Cs-WO_3_ samples due to the appearance of the new shoulders. Therefore, these estimated values of band energies for Cs-WO_3_-0.1 (2.38 eV) and Cs-WO_3_-0.3 (2.47 eV) were obtained from the slopes, which decreased by 0.43 eV and 0.34 eV, respectively, compared to 2.81 eV of WO_3_-0.

### 2.3. Photocatalytic Activity

**Figure 7:**
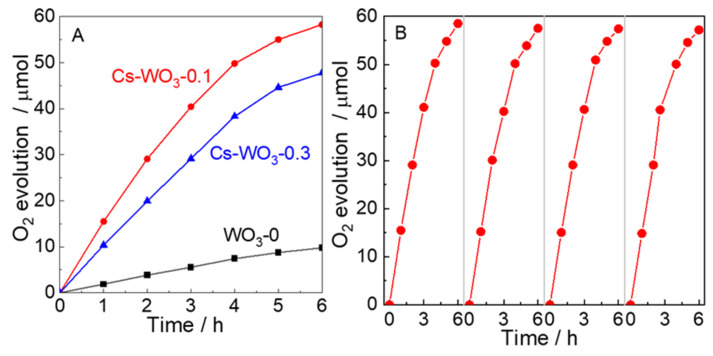
(**A**) Photocatalytic O_2_ evolution over (black) WO_3_-0, (red) Cs-WO_3_-0.1 and (blue) Cs-WO_3_-0.3 (10 mg of catalysts added into 30 mL solution under visible-light irradiation.); (**B**) photocatalytic O_2_ production stability of Cs-WO_3_-0.1.

The photocatalytic O_2_ evolution activities over WO_3_-0, Cs-WO_3_-0.1 and Cs-WO_3_-0.3 were carried out under visible-light irradiation in Fe_2_(SO_4_)_3_ solution; the results are shown in Figure 7. In Figure 7A, the WO_3_-0 generates only 9.76 µmol of O_2_ evolution due to the high recombination rate of photogenerated carriers. It is noted that the activities for O_2_ evolution are drastically improved after Cs doping. The Cs-WO_3_-0.1 shows a higher amount of O_2_ evolution (58.28 µmol), which is an increase of about 6 times and 1.2 times (47.68 µmol) compared with WO_3_-0 and Cs-WO_3_-0.3. It can be explained by the formation of oxygen vacancies, leading to the acceleration of electron and hole transport rates caused by Cs doping. However, the photocatalytic activity decreased with further increases in doping concentration, demonstrating that a higher Cs doping content could also form the recombination centers for the photogenerated carriers caused by excessive dopants. As shown in Figure 7B, the stability test of photocatalytic O_2_ evolution activity for Cs-WO_3_-0.1 was performed repeatedly for four cycles. The results suggest that the Cs-WO_3_-0.1 can be used as an efficient and stable visible-light excited photocatalyst for photocatalytic O_2_ evolution. The small decrease in the O_2_ production rate during the recycling reaction is mainly attributing to the small loss of catalysts during the photocatalytic process at a low dosage of the catalysts, and the powdered catalyst in aqueous solution can be dispersed easily to be taken away from the photocatalytic system during the real-time sampling procedure.

### 2.4. Photoelectrocatalytic Properties

**Figure 8:**
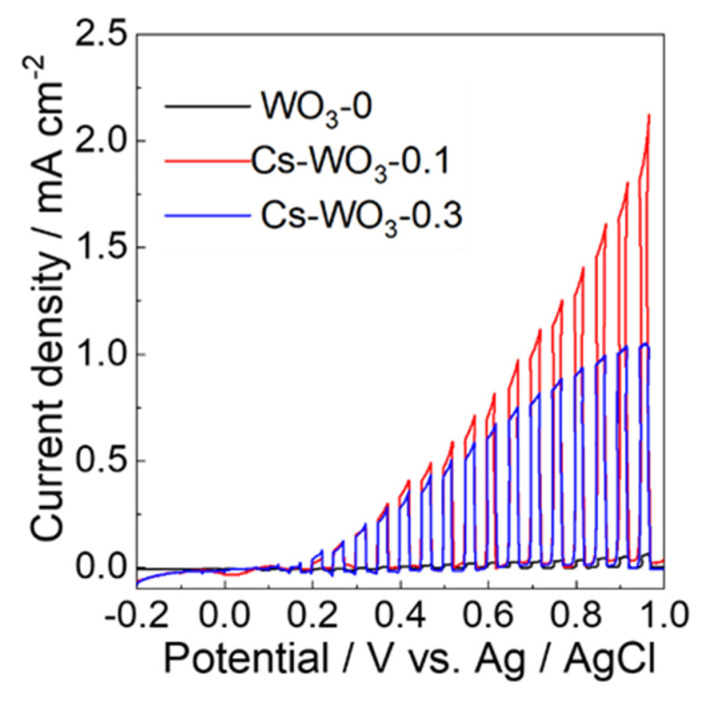
Linear sweep voltammograms (LSVs) of the (black) WO_3_-0, (red) Cs-WO_3_-0.1 and (blue) Cs-WO_3_-0.3 electrodes with visible-light irradiation chopped in a 0.1 M phosphate-buffered solution of pH 6.0 with visible-light irradiation (λ > 420 nm, 100 mW cm^−2^).

The linear sweep voltammograms (LSVs) for these electrodes were taken with chopped visible-light irradiation to investigate their photoelectrocatalytic performances, as shown in Figure 8. The photoanodic currents of these electrodes were observed above 0.1 V vs. Ag/AgCl based on water oxidation. For the WO_3_-0 electrode, the photocurrent of 0.06 mA cm^−2^ at 1.0 V was hard to observe; however, the PEC water oxidation performance of Cs-WO_3_ electrodes was significantly enhanced. The highest photocurrent of 2.12 mA cm^−2^ for Cs-WO_3_-0.1 was generated, which was about two times higher than that of Cs-WO_3_-0.3 (1.1 mA cm^−2^). 

**Figure 9:**
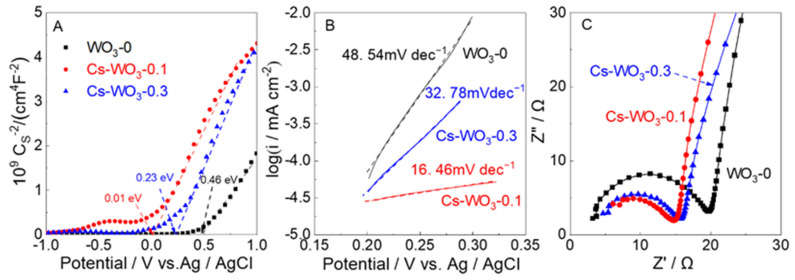
(**A**) Mott–Schottky plots of the (black) WO_3_-0, (red) Cs-WO_3_-0.1 and (blue) Cs-WO_3_-0.3 electrodes in a 0.1 M phosphate-buffered solution of pH, 6.0; frequency, 0.1 Hz; amplitude potential, 10 mV. (**B**) Tafel plots and (**C**) Nyquist plots of the (black) WO_3_-0, (red) Cs-WO_3_-0.1 and (blue) Cs-WO_3_-0.3 electrodes for photoelectrocatalytic water oxidation in a 0.1 M phosphate-buffered solution (pH = 6).

Mott–Schottky plots (Figure 9A) from alternating-current impedance measurements were taken to reveal the relative positions of the valence band (VB) and conduction band (CB) in WO_3_-0 and Cs-WO_3_. As a result, the flat band (E_FB_) potentials (vs. Ag/AgCl) of WO_3_-0, Cs-WO_3_-0.1 and Cs-WO_3_-0.3 were 0.46 eV, 0.01 eV and 0.23 eV, corresponding to 0.66, 0.21, and 0.43 eV (vs. NHE), respectively. The CB potential of the semiconductor material was 0.1–0.3 eV lower than that of the E_FB_ (vs. NHE) [43], so the CB values of WO_3_-0, Cs-WO_3_-0.1 and Cs-WO_3_-0.3 were calculated as 0.56, 0.11 and 0.33 eV (vs. NHE), respectively. The VB values of WO_3_-0, Cs-WO_3_-0.1 and Cs-WO_3_-0.3 were estimated as 3.37 eV, 2.49 eV, and 2.8 eV, respectively, according to the formula of E_g_ = E_VB_-E_CB_ [44]. Moreover, the donor carrier densities (N_D_ [cm^−3^]) were provided from the x-intercept and the slopes of the straight line [45] (Table 2). The N_D_ values of the Cs-WO_3_ were higher than those for WO_3_-0. In particular, the highest N_D_ value for Cs-WO_3_-0.1 (3.46 × 10^19^ cm^−3^) was calculated, which was 1.5 and 1.1 times higher than those of WO_3_-0 (2.26 × 10^19^ cm^−3^) and Cs-WO_3_-0.3 (3.13 × 10^19^ cm^−3^). The negative shift in the E_FB_ potential and the increase in the N_D_ are beneficial to enhance the photocatalytic activity and photoelectrocatalytic performance for the OER.

The Tafel plots are useful to investigate the reaction kinetics of the OER. As shown in Figure 9B, the Tafel slopes of the Cs-WO_3_ prominently decrease compared with those of WO_3_-0. Cs-WO_3_-0.1 possesses a lower Tafel slope of 16.46 mVdec^−1^ than Cs-WO_3_-0.3 (32.78 mVdec^−1^) and WO_3_-0 (48.54 mVdec^−1^), indicating that Cs doping gives a faster kinetic response in the OER and makes the Cs-WO_3_ catalysts have higher photocatalytic activities for the OER.

The electrochemical impedance was utilized to give an insight into the kinetics of the charge transfer process and to evaluate its effect on photocatalytic O_2_ evolution activity. As seen in the results of the Nyquist plots in Figure 9C, Cs-WO_3_-0.1 exhibited smaller semicircles than WO_3_-0 and Cs-WO_3_-0.3. As is well known, the diameter of the semicircle in the Nyquist plot corresponds to the impedance of the electrode, and the larger the radius, the larger the impedance [46]. This result indicates that the Cs-WO_3_-0.1 has a lower charge transfer resistance and higher separation efficiency for photogenerated electron–hole pairs than other electrodes and inhibits the recombination of photogenerated charges. This is mainly due to the n-type doping of WO_3_ by cesium doping, which injects electrons into the Fermi level, enhances the CB potential and also increases lattice defects and oxygen vacancies in WO_3_, thereby improving the conductivity of the WO_3_. The Tafel and electrochemical impedance results provide favorable evidence for the improvement of photocatalytic activity for the OER.

**Figure 10:**
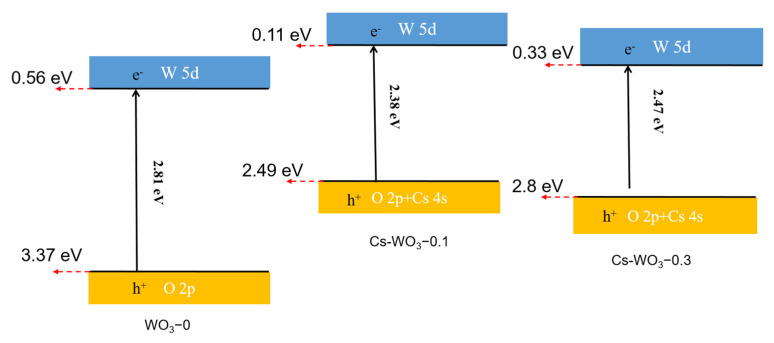
The proposed band natures of WO_3_-0, Cs-WO_3_-0.1 and Cs-WO_3_-0.3.

The energy positions were investigated to elucidate Cs-doping effects on the band energy of WO_3_. Figure 10 shows energy positions for WO_3_-0, Cs-WO_3_-0.1 and Cs-WO_3_-0.3. It has been well documented that the VB of Cs-WO_3_ consists of the hybridization between O2p and Cs 4s, and the CB is from W 5d electronic components. The enhanced optical absorption is illustrated for the contribution from the Cs 4s hybridization in VB.

## 3. Experimental Section

### 3.1. Materials

Tungstic acid (H_2_WO_4_), Hydrogen peroxide (H_2_O_2_), Marpolose (60MP-50), Ethylene glycol (EG, molecular weight = 300), Cs_2_CO_3_ and Fe_2_(SO_4_)_3_·9H_2_O were purchased from Aladdin’s Reagent. A Fluorine-doped tin oxide (FTO)-coated glass substrate was obtained from Dalian HeptaChroma Co., Ltd. (Dalian, China) Millipore water (DIRECT-Q 3UV, Merck Ltd., Shanghai, China) was used for all the experiments. All other chemicals were of analytical grade and used as received unless mentioned otherwise.

### 3.2. Synthesis of WO_3_ Powders

Typically, 1.0 g H_2_WO_4_ (4.0 mmol) was dissolved into the H_2_O_2_ (20 mL) under vigorous stirring at room temperature, forming the pale-yellow solution A. Cs_2_CO_3_ (0.33 g) was dissolved in water to form the B solution. The B solution was added into the A solution dropwise to form the C solution. Then, the C solution was transferred to Teflon-lined stainless-steel autoclaves (reactor volume: 50 mL) at 120–150 °C in 12–36 h for hydrothermal reaction. The 0.1 mol% Cs-doped WO_3_ (Cs-WO_3_-0.1) powder was obtained after centrifugation, washed repeatedly with ethanol, and air-dried. The Cs-WO_3_-0.3 and Cs-WO_3_-0.5 were prepared in the same manner by changing the Cs_2_CO_3_ amounts of 1.0 g and 1.63 g, respectively. A pure WO_3_ sample denoted as WO_3_-0 was prepared in the same manner without the addition of Cs_2_CO_3_.

### 3.3. Fabrication of Electrodes

In the typical procedure, 0.4 g of powder (Cs-WO_3_-0.1 and Cs-WO_3_-0.3) was mixed in the PEG (0.4 mL) with slow stirring until there were no bubbles; a smooth paste was formed. The resulting paste was squeezed over a clean FTO glass substrate by a doctor-blade coater and dried under a 100 W infrared lamp. After repeating the procedure twice, the Cs-WO_3_-0.1 and Cs-WO_3_-0.3 electrodes were prepared. The pure WO_3_ electrode was fabricated by the same method using a precursor prepared without the addition of Cs_2_CO_3_.

### 3.4. Characterization of the Photocatalysts

The crystal structure of the samples was analyzed using an X-ray diffractometer (XRD-6000, Japan) and a scanning speed (4 °C/min). A UV-2700 UV–Vis spectrophotometer (Shimadzu, International Trade (Shanghai) Co., Ltd., Shanghai, China) was used to examine the absorption spectra of solid powder samples. Using a thermal field emission scanning electron microscope, the surface morphology of the samples was examined (SIGMA:500, Jena, Germany). The Energy Dispersive X-ray Spectroscopic (EDS) data were taken using electron probe microanalysis (JEOL JED-2300, Tokyo, Japan) operated at an accelerating voltage of 10 kV. Elemental and valence analyses of the samples were performed using an ES-CALAB Xi X-ray photoelectron spectrometer (manufactured by Thermo Fisher Scientific (China) Co., Ltd., Shanghai, China) and calibrated by the C 1 s peak appearing at 284.2 eV. 

### 3.5. Photocatalytic Activity Measurement

Photocatalytic experiments were conducted in a quartz glass reactor ca. 40 cm^3^, and 10 mg of catalyst was suspended in Fe_2_(SO_4_)_3_·9H_2_O (2.1 mM, 30 mL) solution. Then, the system was degassed by bubbling Ar gas to remove oxygen. Under a 300 W xenon lamp, the photocatalytic process was carried out under continuous stirring to ensure the catalyst dispersed in the solution well. The evolution amount of oxygen was detected by gas chromatography with a TCD detector (Shimadzu GC-8A with a TCD, 5 A column, Ar as carrier).

### 3.6. Photoelectrocatalytic Property Measurement

The photoelectric properties of the samples were tested on a Chenhua CHI760E electrochemical workstation (Shanghai Chenhua Instrument Co., Ltd., Shanghai, China) using a three-electrode system: the FTO photoelectrode as the working electrode (with an approximate working area of 1 cm^2^), platinum wire as the counter electrode, and saturated AgCl electrode (Ag/AgCl) as the reference electrode. 

For photovoltaic performance testing, a 300 W xenon lamp was used (Optical Module X; Ushio Inc., Tokyo, Japan) to simulate sunlight and its light intensity was adjusted to 100 mW cm^−2^. The linear sweep voltammograms (LSVs) were measured at a scan rate of 5 mV s^−1^. Light was irradiated from the back side of the working electrode using a 300 W xenon lamp with a UV-cut filter (*λ* ≥ 420 nm). Electrochemical impedance spectra were measured at an applied potential of 0.68 V vs. Ag/AgCl (1.23 V vs. RHE) in a frequency range of 10 mHz to 20 kHz (amplitude of 50 mV). Light irradiation (*λ* ≥ 420 nm) was conducted with the electrode with a 300 W xenon lamp (Ushio Inc., Tokyo, Japan, Optical ModuleX).

## 4. Conclusions

The Cs-doped WO_3_ with spatial charge separation was synthesized using a hydrothermal approach, which exhibited a significant enhancement of the photocatalytic performance for water spitting to boost the production of oxygen. The addition of Cs dependence on the physiochemical properties and the performance of the photocatalytic OER of the WO_3_-0 and Cs-WO_3_ catalysts were investigated to characterize Cs doped into the WO_3_ lattice and reveal the mechanism of superior performance for the photocatalytic OER of Cs-WO_3_. The Cs doping is responsible for the significant red shift in the absorption edge, with a new shoulder appearing at 440–520 nm compared to that in WO_3_-0. The Cs-WO_3_ catalyst is able to utilize visible light at longer wavelengths below 520 nm for photocatalytic OER, in contrast to utilization below 440 nm for the WO_3_-0 catalyst. These results demonstrate that Cs doping is an effective strategy for improving the photocatalytic performance of WO_3_ photocatalysts for the OER, and thus it is expected to be applied for photocatalytic OERs as artificial photosynthesis to improve the solar energy conversion efficiency.

## Figures and Tables

**Table 1:** Summary of physicochemical properties of different WO_3_ samples.

Samples	Molar Ratio of Cs/W ^(a)^	Crystallite Diameter ^(b)^ (nm)	Lattice Parameters			Surface Area ^(c)^ (m^2^ g^−1^)
			a (Å)	b (Å)	c (Å)	
WO_3_-0	0:1	27.5	7.3125	7.5384	7.5964	9.2
Cs-WO_3_-0.1	0.12:1	25.2	7.2997	7.5009	7.5625	16.1
Cs-WO_3_-0.3	0.15:1	20.9	7.2453	7.4813	7.4953	10.6
Cs-WO_3_-0.5	0.17:1	19.1	7.2046	7.4364	7.4678	8.9

^(a)^ The content of Cs^+^ was analyzed according to the approach we reported previously. ^(b)^ The crystallite diameters were calculated from XRD data according to the Scherrer equation and expressed as average values calculated based on the (311) peak. ^(c)^ The surface areas were provided from N_2_ sorption isotherms.

**Table 2:** Summary of optical and electrochemical properties and energies of band structures of various WO_3_ samples.

Samples	Absorption Energies	E_FB_	N_D_ (10^19^ cm^−3^)	E_CB_	E_VB_
WO_3_-0	2.81	0.66	3.68	0.56	3.37
Cs-WO_3_-0.1	2.38	0.21	3.78	0.11	2.49
Cs-WO_3_-0.3	2.47	0.43	3.82	0.33	2.80

## Data Availability

The original contributions presented in the study are included in the article, further inquiries can be directed to the corresponding authors.
